# Reestablishment of an algae-bacteria pest model using continuous passage in the laboratory

**DOI:** 10.3389/fmicb.2025.1691537

**Published:** 2026-01-20

**Authors:** Thuy M. Nguyen, W. K. N. L. Abeykoon, Alina A. Corcoran

**Affiliations:** 1 Department of Biology, New Mexico State University, Las Cruces, NM, United States; 2 Department of Civil Engineering, New Mexico State University, Las Cruces, NM, United States

**Keywords:** predatory bacterium, parasite, microalgae, *Nannochloropsis*, FD111, *Pseudobacteriovorax*, *Oligoflexus*

## Abstract

**Introduction:**

The paucity of laboratory models comprising predatory bacteria infecting microalgae hinders our understanding of bacteria that predate or parasitize algae both in aquatic ecosystems as well as algal cultivation systems. In 2015, a novel predatory bacterium was found in open cultivation ponds of *Nannochloropsis salina* located in Las Cruces, New Mexico (NM). This bacterium, “FD111”, caused the crash of large-scale cultures in the field, and transfer of the infection source into healthy *N. salina* cultures in the lab killed them. However, research on this pest has been slow due to difficulties cultivating it under laboratory conditions.

**Methods:**

In this study, we continuously passaged two infection sources of an FD111-like pest collected in the field in 2023 into two cultures of *Nannochloropsis* oceanica, CCAP849/10 and a field-adapted culture of the same isolate, with the purpose of enriching for the pest. We passaged the pest into healthy cultures ten times. At each passage, we collected data on the biomass and health of algae cultures and collected samples for 16S sequencing. We also documented the life cycle of the pest via transmission electron microscopy.

**Results:**

Continued passaging of the infection source to healthy algal cultures was a reliable approach to maintain pest viability in the lab, although it did not help to enrich the pest concentration, likely due to the timing of each passage. Following each passage, infected cultures deteriorated and, after four days, died. Transmission electron microscopy showed that a pest similar to FD111 attached to *N. oceanica* cells, replicated inside the cells, and exited the cells after replication – leaving ghost cells. In this study, the FD111-like bacterium had a rod shape and single flagellum, whereas the originally described FD111 had two distinct phenotypes: a rod and hook shape. The 16S microbial community analysis indicated that the FD111-like bacterium falls within the *Oligoflexales* order, possibly the *Pseudobacteriovorax* genus. Sequencing also revealed that the pest was not dominant within the bacterial community, accounting for as little as 3% of the operational taxonomic units.

**Discussion:**

This paper shows that continuous passage can be an alternative approach to isolation and cultivation of pests, and lays the groundwork for additional research and development on this and similar pests. The finding that cultures crashed with a putative pest of low abundance suggests that this pest could be a keystone species in aquatic ponds. Additional work is needed to determine the mechanisms of infection and inform crop protection strategies.

## 1 Introduction

*Nannochloropsis* is a genus of marine microalgae extensively studied and used for its lipid production (Chua and Schenk, 2017; Palanisamy et al., 2023), including that of eicosapentaenoic acid (EPA) (Ma et al., 2016; Chua and Schenk, 2017; Sá et al., 2020; Piyatilleke et al., 2024). The taxon has many industrial applications, with nutraceuticals being the most prominent (Van Vooren et al., 2012; Taleb et al., 2015; Kim et al., 2021). To meet commercial demands, *Nannochloropsis* is typically cultivated in open ponds, a cost-effective method that requires minimal maintenance (Bellou and Aggelis, 2012; Kawaroe et al., 2015; Fulbright et al., 2018; Cunha et al., 2020; Novoveská et al., 2023). However, this approach leaves algal crops vulnerable to infections by pests, including predatory and antagonistic bacteria (Amin et al., 2015; White and Ryan, 2015; Aiyar et al., 2017; Hotter et al., 2021; Rose et al., 2021; Burgunter-Delamare et al., 2024), which can limit biomass productivity and yield during cultivation or, in certain cases, cause whole crop losses. To develop crop protection strategies targeted at antagonistic and predatory bacteria, we must first establish robust algae-bacteria pest models, which we can then use to describe mechanisms of infection and test different crop protection approaches. At present, the scarcity of established laboratory models for studying algal-bacterial interactions hinders a comprehensive understanding of the mechanisms of infection involved.

To our knowledge, only six algae-bacteria pest models have been established in cultivation-relevant algae strains. These models include *Nannochloropsis* and *Bacillus safensis* (Humphrey et al., 2023); *Nannochloropsis* and *Bacillus pumilis* (Fulbright et al., 2016; Ayshoa Al Gabara, 2017); *Chlorella* and *Vampirovibrio chlorellavorus* (Soo et al., 2015; Park et al., 2019); *Nannochloropsis* and FD111 (Lee et al., 2018); *Monoraphidium* and *Oligoflexus*, and *Scenedesmus obliquus* and *Oligoflexus*. With the exception of the *Bacillus* models, the aforementioned pests have been passaged as mixed infection sources (i.e., dead algae and bacteria), as isolation has not been achieved. Humphrey et al. demonstrated pathogenicity of *B. safensis* on *N. gaditana* when an additional 25% carbon source from marine broth media was provided to support the growth of this pest (Humphrey et al., 2023). This infection was controlled using a bacteriophage, a virus that preys on *B. safensis* cells while sparing the algal cells. Fullbright et al. demonstrated that *B. pumilis* negatively affected *Nannochloropsis salina* cultures by secreting an inhibitory chemical; cross-infection into *Chlorella vulgaris* and *Tetraselmis striata* did not cause the same inhibitory effects (Fulbright et al., 2016). *Vampirovibrio chlorellavorus*, a pathogen of *Chlorella sorokiniana*, first described by Gromov and Mamkaeva (1972) and is commonly found in outdoor cultivation systems where it can cause significant biomass loss in algal cultures (Soo et al., 2015; Attalah et al., 2019; Park et al., 2019; Steichen and Brown, 2019; Steichen et al., 2020). Previous work on this pest used passaged infection sources to describe virulence factors including Type IV secretion systems and other genes related to motility and quorum sensing (Soo et al., 2015; Hovde et al., 2020), but recent passaging efforts of resurrected infections from backfrozen infection sources in our lab was not possible.

Following identification of FD111 in 2015, Lee et al. described the crash phenotype of this organism in *N. salina* to include flocculation, discoloration, and culture collapse; proposed a putative life cycle; and offered a simple approach to chemically treat FD111 infections (Lee et al., 2018). Backfrozen infection sources of FD111 were used to generate repeat crashes in *N. salina* and *N. oceanica* between 2015 and 2018. The use of qPCR at Sapphire Energy with primers designed against FD111, coupled with bleach treatment, allowed the company to effectively manage outdoor cultures of *N. oceanica* during its operation. Cross-infectivity into multiple *Nannochloropsis* strains was demonstrated during 2018 (Starkenburg and Corcoran, 2022) and crash samples were used to generate a draft genome of FD111, which identified it as belonging to the genus *Oligoflexus*, within the *Oligoflexia* class (Mascarenas et al., 2025). However, between 2018 and 2023, we were not able to generate crashes from backfrozen stocks of FD111 infection sources, sparking the recapture of FD111 from the field. To reestablish FD111 as a pest model, we collected infection sources from multiple locations throughout NM and passaged those infection sources into *N. oceanica* in the lab. Parallel work at the Arizona Center for Algal Technology and Innovation identified two strains of *Oligoflexus* able to infect two species of freshwater algae, *Monoraphidium minutum* and *Scenedesmus obliquus* (Tan et al., 2025). In that lab, cultures were passaged similarly - from backfrozen or cold-stored infection sources.

Here, we describe the continuous passage of an FD111-like bacterium from two infection sources collected in the field. Specifically, we track infection dynamics through 10 successive passages and complement those data with transmission electron microscopy and 16S sequencing. This work documents the predation of two cultures of *N. oceanica* by an FD111-like pest and shows that passaging mixed infection sources helps to maintain a viable infection source; however, this approach showed limits with respect to enrichment.

## 2 Materials and methods

### 2.1 Algae cultures and cultivation medium

In this study, we used two different cultures of *Nannochloropsis oceanica*: CCAP 849/10 from the Culture Collection of Algae and Protozoa (NCBI BioSample ID: SAMN39538505) and P7C12, a field-adapted culture from Sapphire Energy Inc., where it had been cultivated outside for over 2 years (NCBI BioSample Accession number: SAMN30478310). This culture was originally acquired as CCAP 849/10 in 2015 and maintained outdoors until 2017. P7C12 was suspected to be more resistant to bacterial infections, given its exposure to pests outdoors. Average Nucleotide Identity (ANI) analysis (Rodriguez-R and Konstantinidis, 2014) revealed a 99.95% genetic similarity between P7C12 and CCAP849/10 genomes. Since genomes of the same species typically show ANI values exceeding 95%, these results confirm both CCAP849/10 and P7C12 as *N. oceanica*. However, it was hypothesized that P7C12 had a more supportive microbial community and greater resilience to predatory bacteria, given its culture history outside. Prior to and during experiments, both cultures were grown in 16NFL101:150/40 medium, consisting of the following ingredients per liter of Milli-Q deionized water: 0.82 g sodium bicarbonate (NaHCO_3_), 16 g sodium chloride (NaCl), 0.59 g potassium chloride (KCl), 2.3 g magnesium sulfate heptahydrate (MgSO_4_*7H_2_O), 0.05 g calcium chloride (CaCl_2_) anhydrous, 0.354 mL urea ammonium nitrate solution 32 (UAN-32), 0.288 mL 8.5% phosphoric acid (H_3_PO_4_ v/v), 0.06 mL 100X CM Trace-Fe, and 0.024 mL of a 10,000X Fe Stock (Lee et al., 2018). The 100X CM Trace-Fe solution was prepared by dissolving 50 g of EDTA tetrasodium tetrahydrate salt (4Na-EDTA*4H_2_O), 4.58 g of manganese (II) chloride (MnCl_2_) anhydrous, 2.09 g of zinc chloride (ZnCl_2_) anhydrous, 1.26 g of sodium molybdate dihydrate (Na_2_MoO_4_*2H_2_O), and 0.4 g of cobalt (II) chloride hexahydrate (CoCl_2_*6H_2_O), then diluting the mixture to 1 L with Milli-Q Deionized Water. The 10,000X Fe Stock was made by combining 336.3 g of EDTA tetrasodium tetrahydrate salt (4Na-EDTA*4H_2_O) and 100 g of Ferix-3 and then topping off the solution to 1 L with Milli Q Deionized Water. Cultures were kept in an incubator with 2% CO_2_ at 27.5 °C under continuous light (150 μmol/m^2^/s) provided by 4 16 W LED bulbs (Philips Lighting Inc., New Jersey, United States) and shaken at 90 rpm on a VWR Advanced 5,000 Digital orbital shaker (VWR International Inc., Pennsylvania, United States).

### 2.2 Pest sources

We used two different sources of pests. The first source, known as NMP1, was collected from an open algal cultivation pond in NM that had been infected by an FD111-like bacterium and brought to the lab in May 2023. This source contained a mix of bacteria and other pests (e.g., ciliates, amoeba, and flagellates). The second source, NMP6, was collected from mini ponds located in Las Cruces, NM (Fabian Garcia Research Center: latitude 32.28, longitude: −106.77) at the same time. NMP6 was also a mixed pest source but underwent both passage and filtration through a 0.45 μm pore size membrane to eliminate larger pests but retain the FD111-like bacterium, which measured approximately 0.25 μm in diameter and 2–3 μm in length. We flash froze aliquots of NMP1 and NMP6 in liquid nitrogen and stored them in 1.8 mL cryopreservation tubes at −80 °C until use. These samples remained in the freezer for about a year before commencing this experiment. Once removed from the freezer, the samples were left at room temperature in dim light to thaw before being used to infect the healthy algal cultures.

### 2.3 qPCR analysis to verify FD111 presence in the backfrozen stocks

NMP1 and NMP6 were part of a larger sample set collected during a previous project aimed to identify pests, including FD111 and a golden flagellate, in algal cultivation ponds located in NM and Imperial, TX. When these samples were collected, qPCR was performed at Los Alamos National Laboratory to quantify pest concentrations. About 2 mL of pond samples were collected in duplicate, centrifuged and preserved in RNA later for DNA extraction. DNA was extracted by using Zymo fungi/bacterial Miniprep kit #D6005. Pond lysate was diluted 1:20 in deionized water to obtain genomic DNA. qPCR reactions for the QuantaBio Q System (Bio-Rad) were assembled as follows: 10 μL of SYBR Green 2X SuperMix, 1 μL each of 5 μM forward and reverse primers, 2 μL of template DNA at 5 ng/μL (10 ng total), and 6 μL of nuclease-free water. Serial dilutions were used to detect the target pest, with reactions containing between 2 and 20 ng of total DNA.

Detection of FD111 was achieved using the forward primer GCCAGTCCCAGCTCTTTATT paired with reverse primer CTGCATACCAACGCCTATGA (Award #DE-EE0008902 to the New Mexico Consortium, 2024). For *N. oceanica*, we employed the primer combination GGGAGGAAGGGTGTTTCTTT (forward) with ATGAGGGTGCGGAGATTTATG (reverse). Golden flagellate detection utilized CACGAGCCCGACATCTTATG as the forward primer and CGGATCGGAAGTCAGTTGATAC as the reverse primer. We did not track pest abundance using qPCR during the continuous passaging described in this current study.

### 2.4 Enrichment of FD111-like bacteria

We conducted 10 successive passages of NMP1 and NMP6 into healthy cultures of CCAP849/10 and P7C12, aiming to enrich and selectively enrich for the FD111-like bacteria over other bacteria present in the mixture. Co-cultures were maintained in 100 mL tissue culture flasks equipped with polypropylene screw caps vented by 0.2 μm membranes (MTC Bio Inc., New Jersey, United States), under the culture conditions described above. At each passage, 45 mL of algae at an exponential growth stage with an optical density (OD_750_) of 0.1 was mixed with 5 mL of the infection source, resulting in a 10% v/v infection rate. Triplicate cultures were maintained. As inoculated cultures began to decline (as defined by declines in Fv/Fm, loss of color, and increased flocculation) aliquots were transferred into healthy cultures with a OD750 of 0.1 or 0.2. This initial OD varied depending on the availability of starting culture.

Daily, a 350 μL sample was transferred to a 96-well flat bottom black plate (Corning Inc., Maine, United States). The sample was then allowed to acclimate in the dark for 15 min. Using pulse amplitude modulation fluorescence (Mini-PAM 362 II, Walz, Germany), the photosynthetic yield of the dark-acclimated samples was measured. Fv/Fm is the maximum potential efficiency of light absorption by photosynthetic system II (PSII) and is an indicator of plant stress. The lower Fv/Fm values show impaired photosynthetic performance and potential damage to the PSII complex. After measuring photosynthetic yield, a 100 μL sample was diluted with Milli Q deionized water at a ratio of 1:1 for OD_750_ measurements and 1:20 for chlorophyll fluorescence measurements. OD_750_ and chlorophyll fluorescence (430 nm excitation, 685 nm emittance) were measured using a microplate reader (SpectraMax M2, Molecular Devices, United States).

### 2.5 Transmission electron microscopy

We used transmission electron microscopy (TEM) to characterize the infection course and life cycle of the FD111-like bacterium. On days 0, 2, and 4, we collected 5 mL samples in 20 mL glass scintillation vials (Wheaton Inc., New Jersey, United States). Samples were fixed in 2.5% glutaraldehyde in 0.1 M sodium cacodylate buffer (pH 7.4) with three consecutive washes using a rotary mixer for 10 min per wash and stored at 4 °C prior to processing. Secondary fixation was performed with 2% osmium tetroxide in aqueous solution for 2 h, followed by three 10-min washes with deionized water. Dehydration was carried out through a graded ethanol series (30, 50, 70, 90, and 100%), with the final 100% ethanol step repeated twice. A 1:1 mixture of 100% ethanol and Spurr’s resin was prepared for sample infiltration for 1 h, after which samples were transferred to 100% Spurr’s resin for an additional 1-h infiltration. Samples were embedded in resin blocks and polymerized at 60 °C for 48 h. Embedded material was sectioned at 70 nm using a diamond knife (Diatome, Switzerland) on a Leica UC6 ultramicrotome (Leica, Germany). Sections were collected on 200-mesh copper grids, stained with 2% uranyl acetate for 20 min, washed with deionized water, counterstained with 3% lead citrate for 10 min, and washed again with deionized water. Sections were imaged at 80 kV on a Hitachi H7650 transmission electron microscope (Hitachi, Japan).

### 2.6 16S microbial community analysis

We used 16S microbial community analysis to capture a snapshot of the bacterial composition during the time course of the algae culture crash. In addition, we aimed to compare the microbial communities of the *N. oceanica* lab culture with the field-adapted P7C12 culture, which carried a diverse microbial community. A 2 mL sample from passage 10 was snap frozen in liquid nitrogen and stored at −80 °C for subsequent DNA extraction. The samples were sent to the Microbiome Sequencing Service: 16S Amplicon Sequencing (Zymo Research, Irvine, CA) for processing and analysis. The DNA was extracted using the ZymoBIOMICS®-96 MagBead DNA Kit (Zymo Research, Irvine, CA) on an automated platform. Bacterial 16S ribosomal RNA gene sequencing was performed with the Quick-16S™ NGS Library Prep Kit (Zymo Research, Irvine, CA) targeting the V3-V4 region of the gene. This library preparation process involved real-time PCR reactions to control cycles and minimize PCR chimera formation. The resulting PCR products were quantified with qPCR fluorescence readings and pooled based on equal molarity before being cleaned and concentrated with the Select-a-Size DNA Clean & Concentrator™ (Zymo Research, Irvine, CA). Final quantification was done with the TapeStation® (Agilent Technologies, Santa Clara, CA) and Qubit® (Thermo Fisher Scientific, Waltham, WA) systems before sequencing on an Illumina® Nextseq™ with a P1 reagent kit (600 cycles). To improve sequencing accuracy, 30% PhiX spike-in was used during the sequencing process.

The raw data was processed using the DADA2 pipeline which included demultiplexing, joining pairs, and filtering for quality based on the q-score (Callahan et al., 2016). Next, the QIIME 2 pipeline was applied to generate a table of features and select representative sequences (Bolyen et al., 2019). Taxonomic analysis at order level was performed using QIIME 2 with the SILVA 138.2 SSURef NR99 full-length database focusing on the V3-V4 region of 16S rRNA gene. Visualization of key taxa was done in Primer-e (v7). Specifically, we reduced the species set, keeping the 15 most important species, we performed a square root transformation to create a resemblance matrix, standardized samples and performed hierarchical clustering. A shade plot was generated on square root transformed data. We also constructed amplicon sequence variant (ASV) abundance heatmap at species level using unique amplicon sequences derived from raw sequencing data. We employed DADA2 software to distinguish sequences differing by just a single nucleotide, and to examine distribution patterns beyond standard taxonomy assignments. We applied hierarchical clustering to samples using Bray–Curtis dissimilarity metrics and similarly clustered taxa to group those with comparable distribution patterns (Cormack, 1971; Everitt, 1980).

## 3 Results and discussion

### 3.1 Enrichment of the FD111 infection source

Quantitative PCR conducted on field cultures prior to this study showed that FD111 or an FD111-like bacterium was dominant in both NMP1 and NMP6 field samples (Supplementary Figure S1). The qPCR analysis focused on two pests of *Nannochloropsis*: FD111 and a golden flagellate known to prey upon algae in other cultivation systems. Cycle threshold values below 20 for FD111 in NMP1 and NMP6 indicated high pest abundance, whereas high Ct values for *Nannochloropsis* suggested poor algal culture health in these NM ponds. By comparison, Texas cultivation ponds exhibited significantly higher cycle threshold values for FD111, demonstrating that while the pest was present, it had not yet negatively affected cultivar health in these locations. These data show that FD111 or an FD111-like bacterium (i.e., a bacterium that would be detected by the FD111 primers) was present in samples at the time of collection.

**Figure 1:**
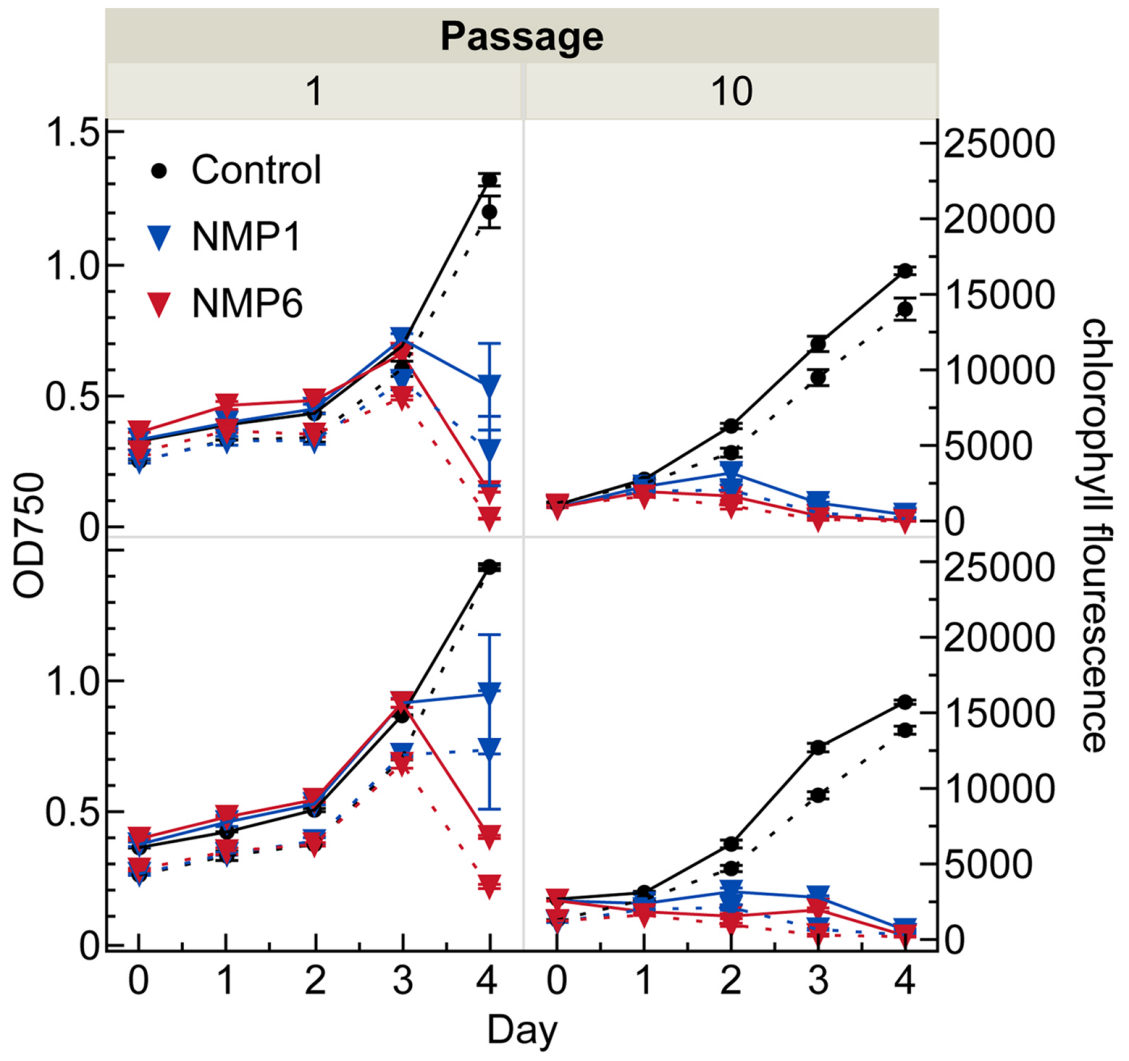
Optical density (left y-axis) and chlorophyll fluorescence (secondary right y-axis) of the *N. oceanica* lab (top, CCAP 849/10) and field-adapted (bottom, P7C12) cultures crossed with two bacterial infection sources, NMP1 (non-filtered sample) and NMP6 (filtered sample). The solid lines show OD750, whereas the dashed lines show chlorophyll fluorescence.

**Figure 2:**
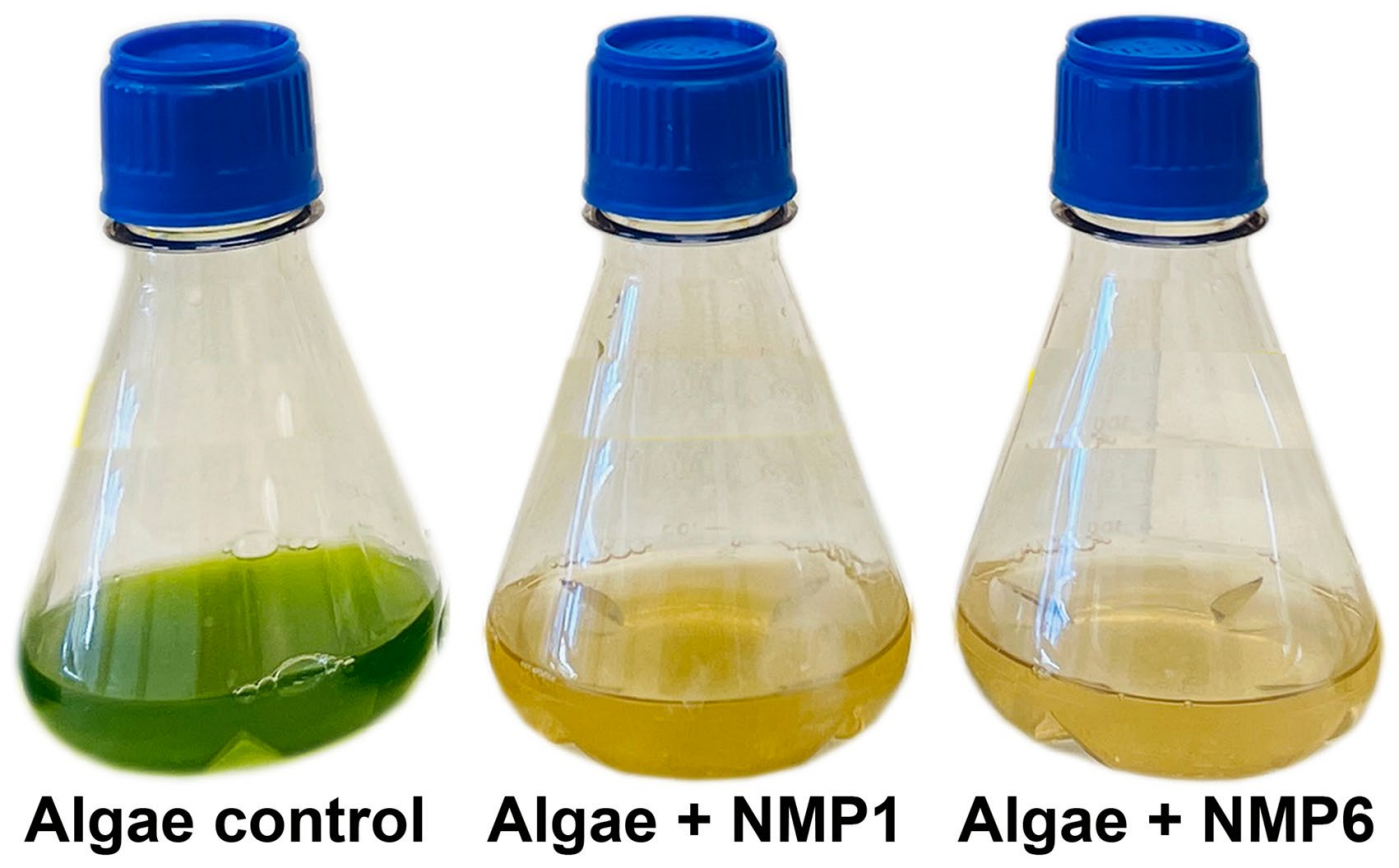
The algae in the control group remained green, whereas the algae with either NMP1 or NMP6 infection turned yellowish after 4 days of culturing.

In the first passage, the addition of NMP1 and NMP6 caused the cultures to crash after 4 days, as evidenced by OD_750_ and chlorophyll fluorescence data (Figure 1), as well as a notable visual shift in the culture’s appearance (Figure 2; Supplementary Figure S2). Infected cultures shifted from a vibrant green color to a yellow/brown color due to chlorophyll degradation. In passage 1, cultures infected with NMP6 were lost quicker than those infected by NMP1 (Figure 1, left). By the 10th passage, the pests caused crashes in both algal cultures prior to day 4 and at the same rate (Figure 1, right). In fact, at this point, growth of the cultures was impeded following infection. Two possibilities can explain this change in crash rates through time: (1) different microbial communities present in the infection sources at passage one and passage 10 (see 3.3. below), or (2) the different starting optical densities of the passages. Previous work has shown that cultures algal cultures at lower cellular densities are affected by bacterial pests easier than cultures at higher optical densities. Successive passaging occurred over the course of 6 weeks, with each crash occurring within 4 days, but as short as 1 day post infection (Supplementary Figure S3). To our knowledge, this is the first published report of successive passaging of a pest from the aforementioned pest models.

Both cultures, CCAP849/10 and P7C12, were affected by the addition of infection sources, despite having different microbial communities. This finding was contrary to our hypothesis as we suspected that P7C12 would be resistant to infection, either via evolution of traits relevant to resistance (e.g., cell wall thickness) because of its outdoor cultivation history or that the unique microbiome would have conferred a protective advantage. Neither was the case, demonstrating that pest resistance had not yet evolved in P7C12 and that the FD111-bacterium was not affected by the associated bacteria in P7C12.

### 3.2 Microscopy

**Figure 3:**
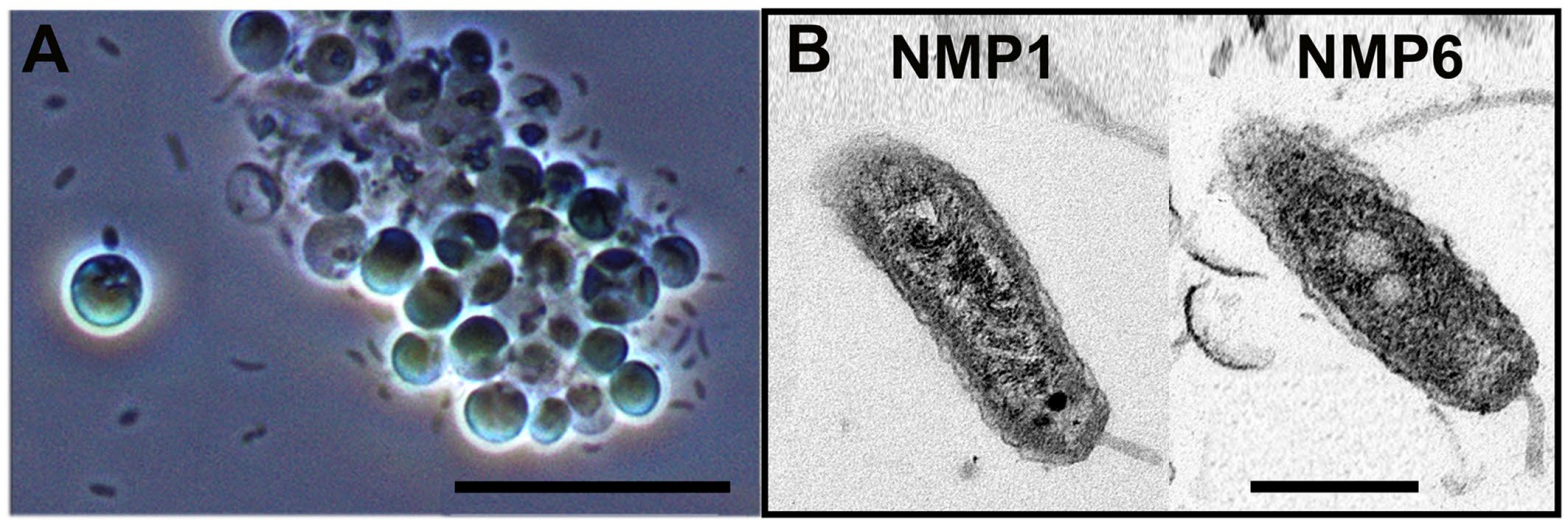
**(A)** Light microscopy images of *N. oceanica* infected by FD111-like bacterium at 10 m. **(B)** TEM images of FD111-like bacterium from NMP1 and NMP6 samples AT 500 nm.

Upon the collapse of the CCAP849/10 and P7C12 cultures, *N. oceanica* cells were seen clumping together under the light microscope. Light microscopy also revealed a dominant bacterium in NMP1- and NMP6- infected algae cultures - a rod-shaped bacterium with little curvature (Figure 3A). There was no discernible difference in the morphology of the dominant bacterium between the two infection sources NMP1 and NMP6. However, this morphology is different than that reported for FD111, which included both hook- and rod- shaped bacteria (Lee et al., 2018). TEM confirmed that the dominant bacterium in NMP1- and NMP6-infected cultures was a rod-shaped bacterium with a flagellum (Figure 3B). FD111 was initially characterized as similar to Bdellovibrio-and-like organisms (BALOs) (Lee et al., 2018). BALOs can shift their morphology between a predatory and a non-predatory phases such that in the predatory stage, they exhibit a vibrioid shape with a polar flagellum, whereas during the non-predatory stage, their cellular shape varies (Seidler and Starr, 1969; Snyder et al., 2002; Davidov and Jurkevitch, 2004). The morphological differences of the FD111-like bacteria observed in this study compared that in Lee et al. alludes to either taxonomic differences between our pest and FD111, environmentally-induced morphological changes; or evolution of predatory traits through time (Snyder et al., 2002). The latter is not likely, given the lack of continuous co-culture of *Nannochloropsis* and FD111 between the time of initial infection (2018) and recapture.

**Figure 4:**
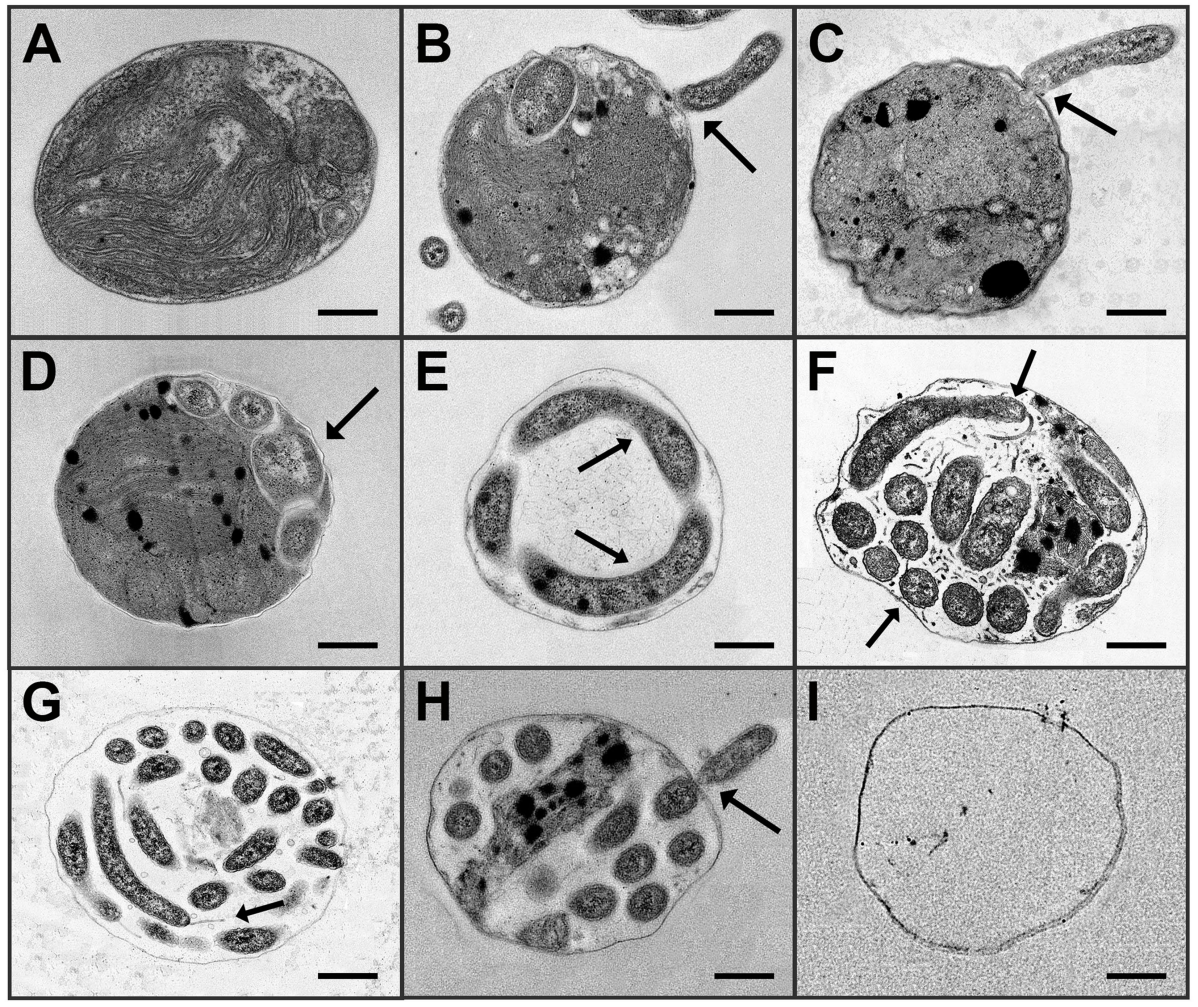
The potential life cycle of an FD111-like bacterium with a *N. oceanica* algal host as observed by TEM is as follows: **(A)** Healthy *N. oceanica* algal cell alone. **(B,C)** FD111-like bacterium attached to the algal cell wall and transferred its cellular material into the host cell through a pore in the wall. **(D)** Early infection: Enlargement within the host. **(E)** Elongation within the host and consumption of the host contents. **(F,G)** Late infection: FD111-like bacteria with flagellum at longitudinal shape, and in a coiled shape. **(H)** FD111-like bacterium exited the algal host cell. **(I)** Algal cell wall is left empty, without any organelles. Scale bar: 250 nm.

TEM images helped describe the potential infection mechanism and life cycle of the FD111-bacterium interacting with the *N. oceanica* algal host (Figure 4). Healthy *N. oceanica* algal cells appeared with intact organelles, a full chloroplast, and a complete cell wall (Figure 4A). With added infection sources, the FD111-like bacteria attached to the cell wall of *N. oceanica* algal (Figure 4B) and appeared to inject material into the cell through what appeared to be a pore in the algal cell wall (black arrow); this injection was coincident with loss of structural integrity of the cell wall (Figure 4C). At this early infection stage, the FD111-like bacteria started reproducing inside host cells with a coiled shape (Figure 4D). The algae organelles were still visible at this time. After that, the bacterial cells continued consuming the host cell organelles and elongated inside host cells (Figure 4E). As the infection progressed, the FD111-like bacteria elongated within cells and segmented (Figures 4F,G). We suspect that the FD111-like bacterium consumed algal cells from the inside out, but further studies are necessary to document this. Toward this end, isotope labeling studies would be helpful. Once the algal organelles were consumed by infected bacteria, the bacteria exited the cells (Figure 4H), leaving empty ghost cells (Figure 4I). This infection and reproduction mechanism is similar that previously proposed by Lee et al. (2018).

### 3.3 Microbial community structure

**Figure 5:**
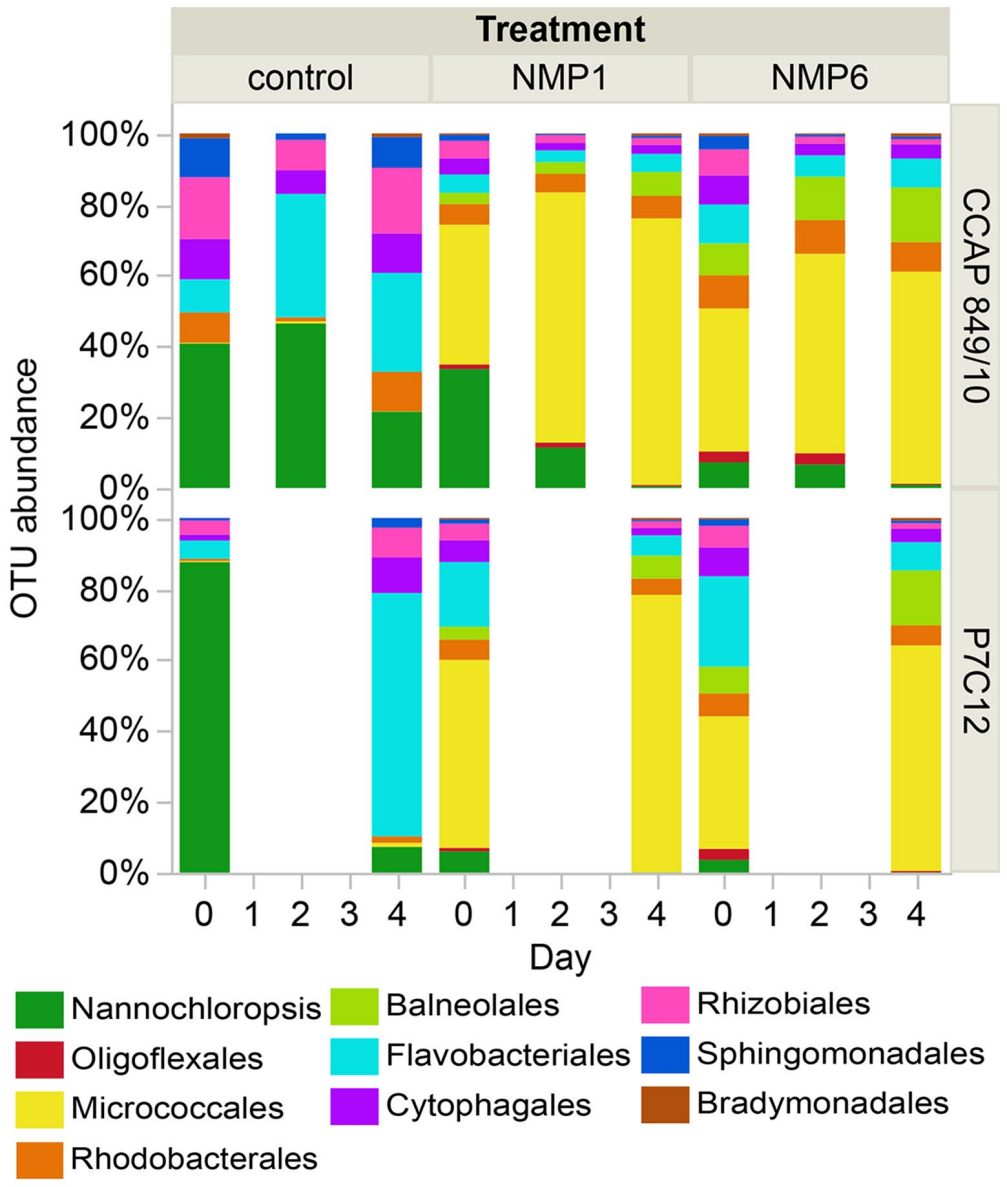
OTU abundance of the top 10 orders in the microbial community for two *N. oceanica* cultures after cross-infection from the two sources over four days. Samples from the lab CCAP 849/10 were taken on days 0, 2, and 4, with duplicates, whereas samples from the field-adapted P7C12 were collected on days 0 and 4, without any replication. Algal chloroplast data are shown in green.

Prior to inoculation with FD111, the microbial communities in the *N. oceanica* lab culture CCAP 849/10 and the field-adapted culture P7C12 differed (Figure 5; Supplementary Table S3; Supplementary Figure S4). In CCAP 849/10, bacteria were more prevalent than algae, whereas in P7C12, algae were the most dominant. Over the 4-day culturing period, the microbial community of the control underwent changes. By day 4 in the control, the most dominant groups included *Nannochloropsis*, Rhodobacterales, Flavobacteriales, Rhizobiales, Sphingomonadales, and Cytophagales (Figure 5). At this point, when the algal culture completely collapsed, the microbiome profiles of the four treatment combinations appeared similar. *Nannochloropsis* was entirely absent in the crashed cultures. The dominant microbiome also shifted with the culture crash, with Micrococales becoming the most dominant order in the treatment. Rhodobacterales, Balneolales, and Flavobacteriales were also observed as dominant groups in the crashed culture (Figure 5). As previous work identified FD111 as *Oligoflexus*, we expected this taxon to be dominant in the NMP1- and NMP6- infected algae cultures. *Oligoflexus* was not detected in any of the samples, but bacteria in the order *Oligoflexales* were present in both the NMP1 and NMP6-infected cultures and absent in the control cultures. The abundance of these organisms was 1.5% on day 0, 3% on day 2, 0.2% at the end of the experiment (day 4). Notably, *Oligoflexales* were not dominant in the co-cultures that crashed. Other studies have documented disproportionately impactful species in species dynamics, across a variety of systems including microbes (Brauner et al., 2025). For example, Brauner et al. (2025) used a co-occurrence network analysis to identify 10 keystone microbes in nearshore aquatic environments. Notably, some of these microbes included parasitic BALOs. Well known keystone bacteria in aquatic systems include SAR11 clades and parasitoid bacteria Bdellovibrio (Brauner et al., 2025). Our results highlight that *Oligoflexales* could have disproportionate negative effects on *Nannochloropsis*, highlighting the importance of this single taxon - a rare taxon - in this algal culture. Alternatively, *Oligoflexales* could be part of a larger functional group of predatory and/or antagonistic bacteria that killed *Nannochloropsis* in our experiments.

**Figure 6:**
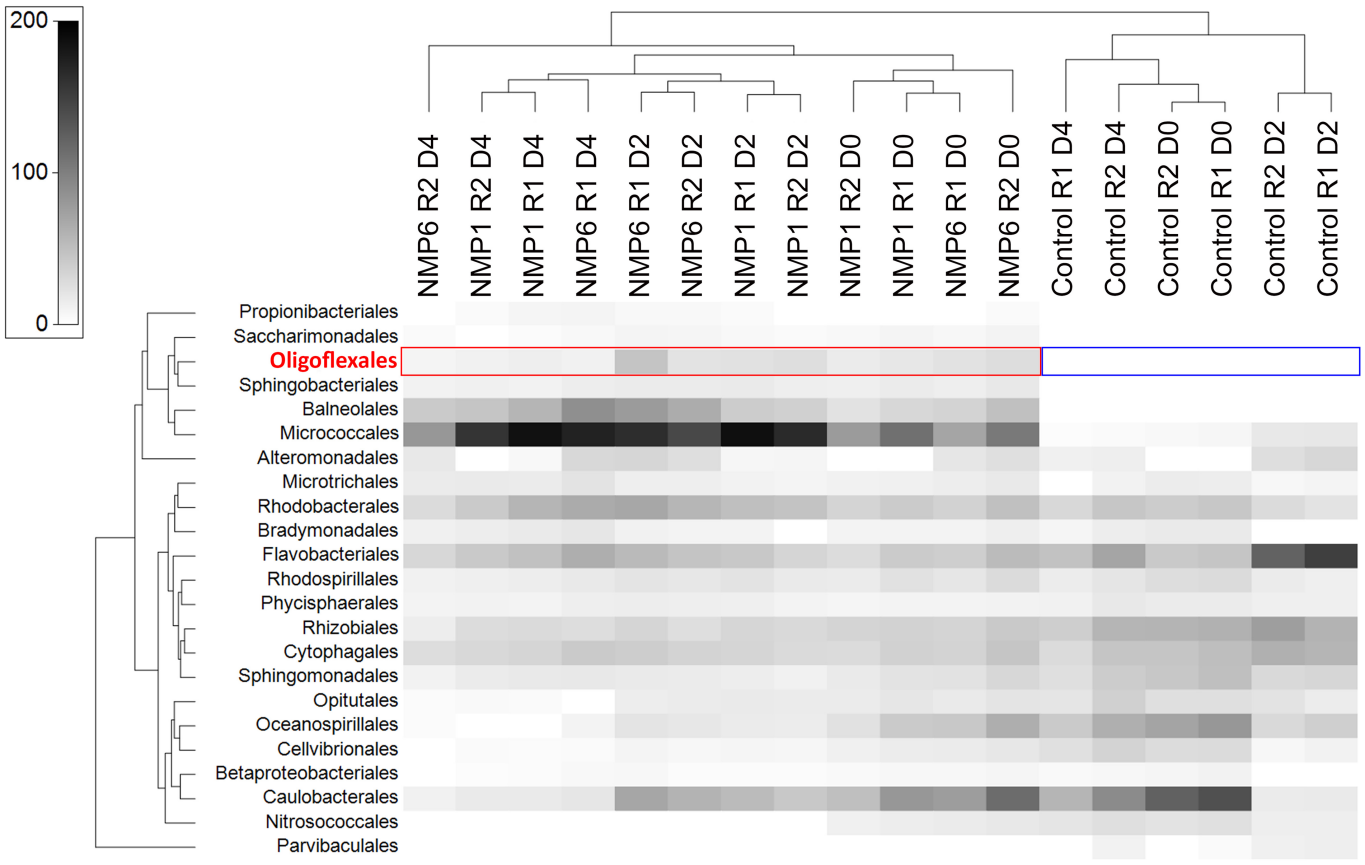
The heat map shows OTU abundance in *N. oceanica* lab culture, including the algae control and those cross-infected with two sources over 4 days. “R” stands for replicate, “D” stands for day. Chloroplast (algae) abundance was not included in the figure.

Removing the algae biomass from our sequence data, we further explored the abundance of bacterial taxa during the time course of our crashes (Figure 6). The control samples clustered together, and the NMP1 and NMP6 treatments at the same day clustered together, showing a stronger temporal signal than pest signal. Micrococcales emerged as the most dominant order in the treatments. Four orders - Propionibacteriales, Saccharimonadales, *Oligoflexales*, and Sphingobacteriales - did not appear in the control but were present in the treatments and during the culture crashes. *Oligoflexales* was absent from the control at all time points, but was found in all treatments, being most abundant in the NMP6-infected CCAP849/10, in a single replicate before the culture crashed. At this point, *Oligoflexales* was 3% of the community. This result was unexpected, as parallel work on *Oligoflexus* in *Scenedesmus obliquus* shows that is the most dominant bacterium during a crash (Tan et al., 2025). The low relative abundance of *Oligoflexus* in our study is curious. If *Oligoflexus* was the sole crash agent in our experiments, this work demonstrates disproportionate effects of a single taxon in affecting the algal population. Still, it is possible that we have multiple crash agents in our infection sources. Next steps on this work include efforts to obtain isolates (e.g., plaque plates, differential centrifugation, filtering and flow cytometry should be used to obtain isolates) to pursue Koch’s postulates. Moreover, additional sequencing would be helpful to better compare our pest with the draft genome of FD111. This work will lay the groundwork for development of targeted crop protection approaches that expand beyond non-specific chemical treatments.

Both CCAP and P7C12 cultures are non-axenic, containing *N. oceanica* with several bacteria. Since *N. oceanica* is the only chlorophyll-containing organism present, increasing chlorophyll levels directly indicate algal growth (Figure 1). Figure 5 shows the relative abundance of algae and associated bacteria through time. The Operational Taxonomic Unit (OTU) percentages reveal shifts in bacterial community composition throughout the experiment. The decrease in the relative percentage of algae does not indicate a population crash but rather reflects faster bacterial growth rates. While algal populations continued to increase from baseline, their proportional representation within the microbial community declined as bacterial populations expanded more rapidly. These patterns were consistent throughout both algae cultures. Controls grew through time, and the infected culture died after 4 days (Figure 2). The algae relative abundance decreased as other bacterial in the polyculture grew more rapidly.

The sequencing reads from both co-culture of *N. oceanica* and two infection sources were blasted again the SILVA 138.2 SSURef NR99 full-length database, specifically targeting the V3-V4 region of the 16S rRNA gene. This analysis identified three species within the Oligoflexaceae family, all classified under the *Pseudobacteriovorax* genus, showing that we have at least three FD111-like variants in our infection sources (Supplementary Tables S1, S2). The only species-level match found in the database was *Pseudobacteriovorax antillogorgiicola*, a gram-negative bacterium, within the order Bdellovibrionales (McCauley et al., 2015; Kieffer et al., 2019). This taxon was collected and isolated in 2025 from the coast of San Salvador, Bahamas. The morphology of *P. antillogorgiicola* was described using TEM as a rod shape with a long curly tail (McCauley et al., 2015). However, the TEM in the current study showed a shorter tail and a more symmetrical body (Figure 3B). In our study, the other two *Pseudobacteriovorax* species found belonged to unculturable taxa, with one identified only at the genus level. There has been little research aimed at isolating these organisms and describing their physiology and infection mechanisms, leaving an opportunity for additional research to further explore this subject.

**Figure 7:**
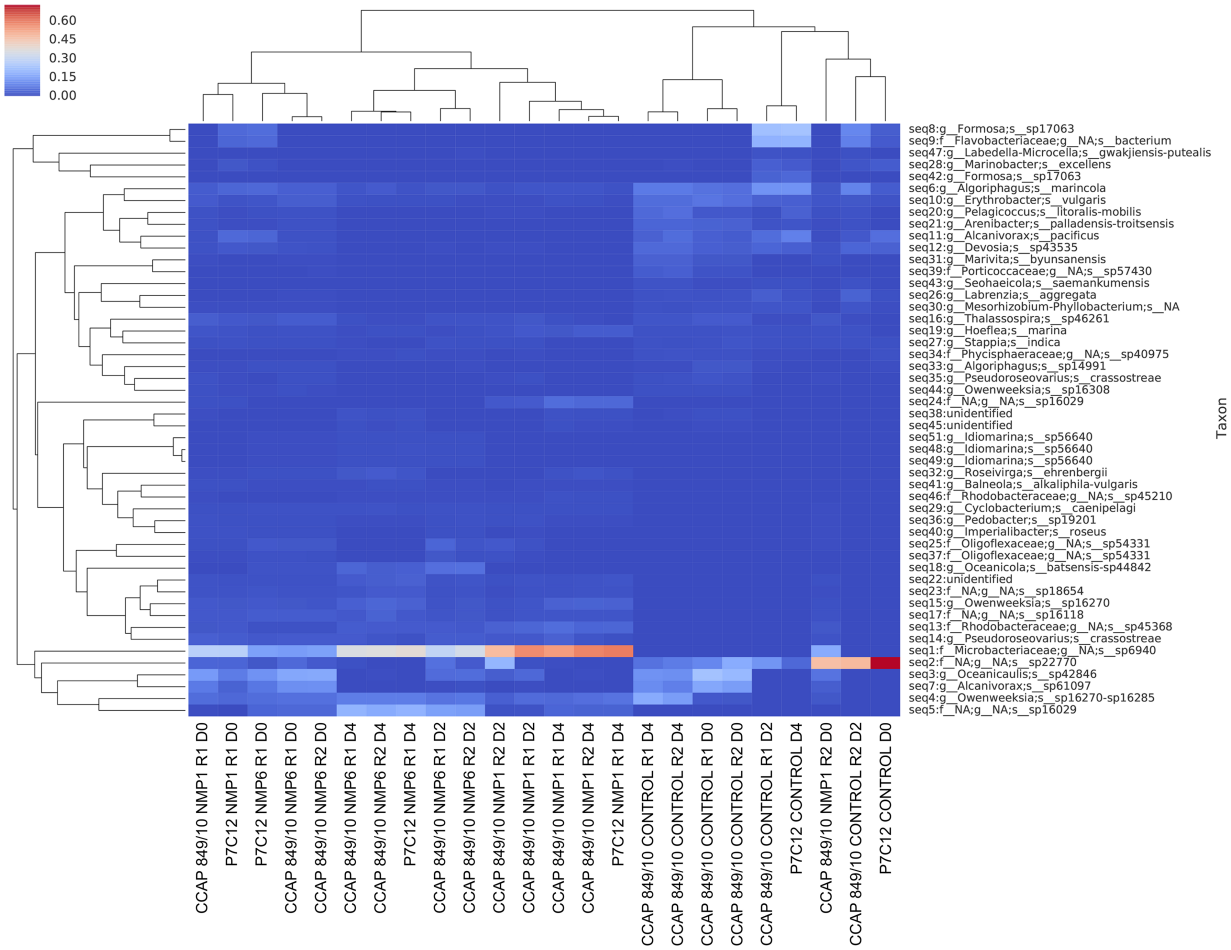
Amplicon sequence variant (ASV) heatmap at sub-species level shows the microbial composition across all samples, highlighting the top 50 most abundant species identified. Rows correspond to individual taxa, while columns represent distinct sample. “R” stands for replicate, “D” stands for day.

We also used DADA2 to construct amplicon sequence variant (ASV) abundance heatmap at sub-species level (Figure 7). The ASV map allowed us to differentiate two similar sequences by just a single nucleotide, showing distinct subspecies only visible through analyzing unique sequence abundance patterns. Figure 7 illustrated microbial composition at sub-species level, featuring the 50 most prevalent species. Each horizontal row displays taxon abundance, whereas vertical columns represent individual samples. The ASV heatmap revealed several beneficial bacteria present only in the control samples that disappeared in the crashed algal culture. *Marinobacter excellens* appeared in high abundance in control samples and at day 0 of the infected culture, where it typically promotes microalgal growth (Cheng et al., 2025; Peña-Montenegro et al., 2023; Chernikova et al., 2020). Similarly, *Algoriphagus marincola* showed strong presence in the control but diminished as the algae deteriorated; this bacterium is known to enhance microalgae thermotolerance by boosting antioxidant enzyme activities and upregulating heat shock protein gene expression (Xu et al., 2023; Huang et al., 2025; Nedashkovskaya et al., 2004). In addition to *Oligoflexales*, we identified other bacteria harmful to algae that proliferated in the crashed culture compared to the control. *Arenibacter* sp., which can eliminate harmful algal blooms, demonstrated selective algicidal activity against various algal species, particularly Pyrrophyta and Ochrophyta. *Pseudooceanicola batsensis* (formerly *Oceanicola batsensis*), a marine bacterium from the Roseobacter clade, exhibits a complex relationship with algae like *Emiliania huxleyi* - initially promoting growth through hormone-like molecules, but potentially causing algal death when bacterial metabolites reach toxic concentrations (Lai et al., 2015). The bacterial family Microbacteriaceae dominated the crashed algal cultures, though current literature has not documented any specific interactions between these bacteria and algae.

## 4 Conclusions and next steps

Despite the importance of microbial interactions in aquatic ecosystems and artificial cultivation systems alike, there are only a handful of laboratory model systems that consist of predatory or parasitic bacteria killing microalgae. There are also a number of systems for which work has started but later halted because of issues maintaining the bacteria-algae systems. One example is that of *Vampirovibrio chlorellavorus* and *Chlorella* (Hovde et al., 2020). Another is FD111 and *Nannochloropsis*. FD111 was originally identified following the crash of industrial scale ponds in 2016 at Sapphire Energy (Las Cruces, NM). A single paper exists on the organism (Lee et al., 2018) as work past this point was hindered because backfrozen infection sources of FD111 were not able to generate the original kill phenotype in *Nannochloropsis*. Our work demonstrates re-capture of an FD111-like pest from the field, with the same life cycle as previously documented, highlighting the ubiquitousness of this pest temporally in NM. It also demonstrates that continuous passage can be one approach to maintain an active infection source within the lab if isolation is not possible or if freezing samples alters the functionality of pests. Finally, it provides a described infection source from which to isolate infective agents. However, continuous passaging was ineffective at enriching pest concentrations. At each passage, we transferred the sick culture on day 4, coinciding with culture crash and yellowing. The timing of this transfer likely influenced our ability to enrich for the pest. Indeed, FD111 will consume *N. oceanica*, reproducing intracellularly, then perish after host cell death. Previous work has shown that such parasite–host relationships create significant population bottlenecks in the parasite population (Papkou et al., 2016); that is, the death of the host in turn causes the death of the parasite. We now suspect that earlier transfers would have enriched the infection source. Future work should identify the precise timepoint of peak bacterial concentration to optimize transfer protocols and maximize infection enrichment.

## Data Availability

The data presented in this study are publicly available. The data can be found here: https://www.ncbi.nlm.nih.gov, accession PRJNA1390289.
