# Supplementary material for: Reestablishment of an algae-bacteria pest model using continuous passage in the laboratory
Source: Front Microbiol. 2026 Jan 20;16:1691537. doi: 10.3389/fmicb.2025.1691537 (PMC12865303; doi:10.3389/fmicb.2025.1691537)
Supplement: Supplementary file 1 [file Data_Sheet_1.PDF]

Continuous Passage of Predatory Oligoflexales Bacteria in *Nannochloropsis oceanica*

Thuy M. Nguyen[1*], W. K. N. L. Abeykoon[2], Alina A. Corcoran[1]

[1]Department of Biology, New Mexico State University, Las Cruces, NM 88003, USA

[2]Department of Civil Engineering, New Mexico State University, Las Cruces, NM 88003, USA

[*]corresponding author: tmnguyen@nmsu.edu

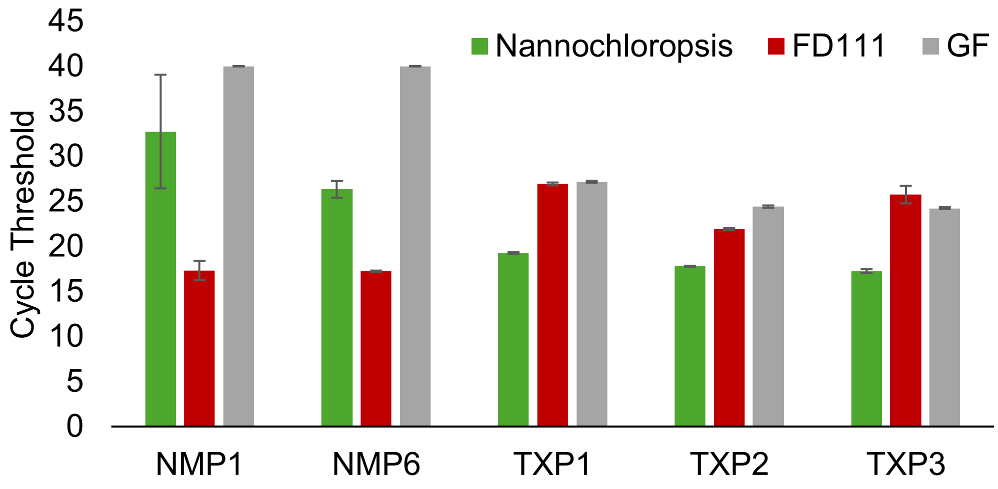

Figure S1. qPCR data from field samples taken from New Mexico Ponds NMP1 and NMP6, as well as Texas Ponds TXP1, 2, and 3. In NMP1 and NMP6, the FD111 bacterium was more prevalent compared to *Nannochloropsis* sp. and golden flagellate (GF), indicated by its lower cycle threshold. Conversely, in the TXP, the FD111 bacterium was less dominant, demonstrated by a higher cycle threshold value.

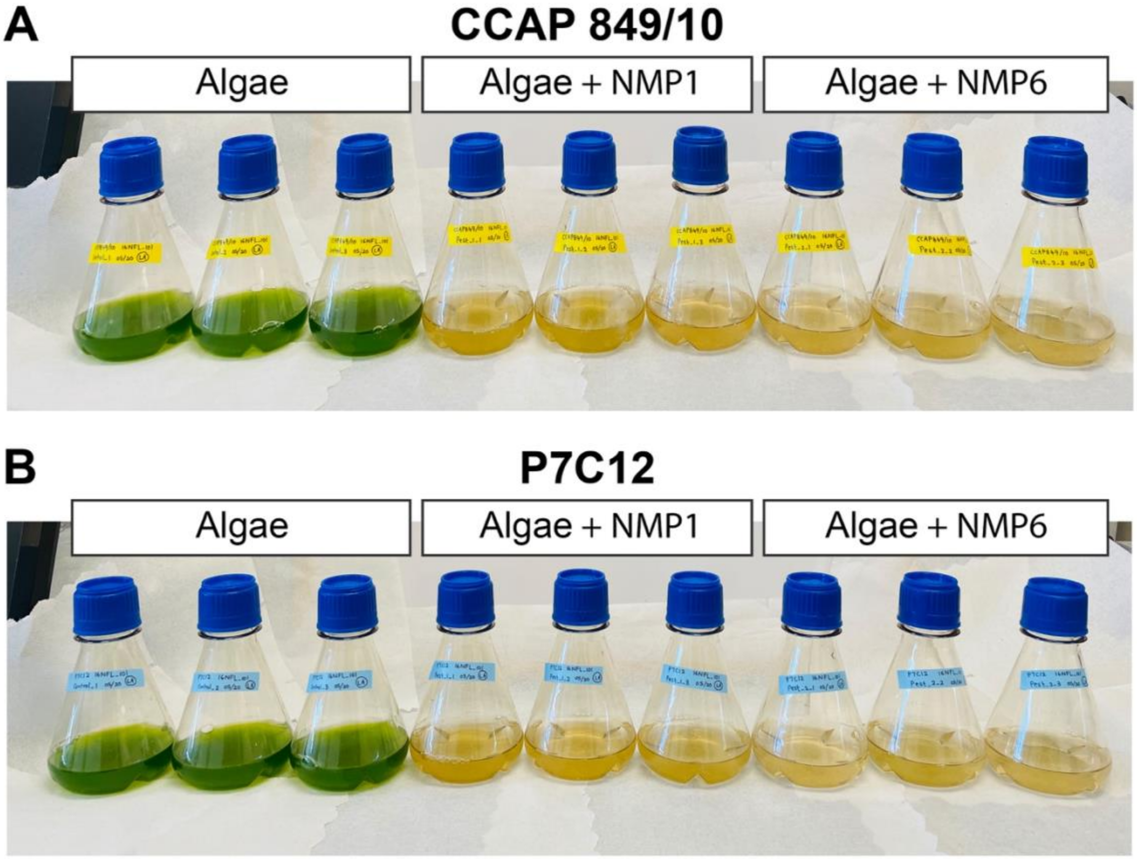

Fig. S2. Flask photos of **A.** CCAP 849/10, and **B.** P7C12. From left to right: algae alone, algae with NMP1, and with NMP6, 3 replicate each treatment.

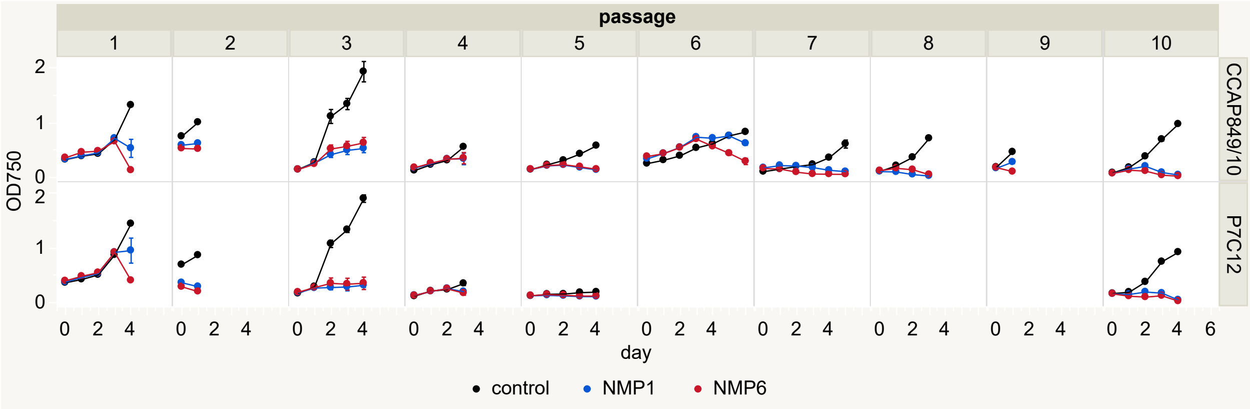

Figure S3. Optical density of 10 passages of two *N. oceanica* cultures (CCAP 849/10 laboratory culture and P7C12 field-adapted culture) crossed with two bacterial infection sources NMP1 and NMP6. *Passages 6 to 9 for the P7C12 culture were absent due to the unavailability of a healthy algal culture.*

Table S1. Statistical analysis to compare field-adapted P7C12 vs lab culture CCAP 849/10

Statistics	Field-adapted culture P7C12	Lab culture CCAP84910
Average Nucleotide Identity (ANI)	99.95%	
# contigs	137	1530
# contigs (>= 0 bp)	137	1530
# contigs (>= 1000 bp)	137	1530
# contigs (>= 5000 bp)	136	765
# contigs (>= 10000 bp)	133	547
# contigs (>= 25000 bp)	127	286
# contigs (>= 50000 bp)	77	153
Largest contig	1674189	686279
Total length	28273855	30127052
Total length (>= 0 bp)	28273855	30127052
Total length (>= 1000 bp)	28273855	30127052
Total length (>= 5000 bp)	28269899	28415727
Total length (>= 10000 bp)	28251839	26878032
Total length (>= 25000 bp)	28154294	22619004
Total length (>= 50000 bp)	26288145	18035050
N50	733541	68738
N90	69104	8864
auN	738527	119998
L50	13	103
L90	63	573
GC (%)	53.97	54.26
**Per base quality**		
# N's per 100 kbp	0.71	15.5
# N's	200	4670

Table S2. The sequencing reads of co-culture of *N. oceanica* and 2 infection sources were blasted again SILVA 138.2 SSURef NR99 full-length database focusing on the V3-V4 region of 16S rRNA gene. This analysis revealed the presence of three species in the Oligoflexaceae family, all belonging to the *Pseudobacteriovorax* genus. The only matching hit in the database was identified as *Pseudobacteriovorax antillogorgiicola* organism at the species level.

**Taxonomy identification**	**Confidence score**
o_Oligoflexales; f_Oligoflexaceae; g_Pseudobacteriovorax; s_uncultured bacterium	0.89
o_Oligoflexales; f_Oligoflexaceae; g_Pseudobacteriovorax; s_Pseudobacteriovorax antillogorgiicola	0.81
o_Oligoflexales; f_Oligoflexaceae; g_Pseudobacteriovorax	0.77

Table S3. The absolute abundance of three different *Pseudobacteriovorax* species in the Oligoflexaceae family

Sample ID	Pseudobacteriovorax sp.	P. uncultured bacterium	P. antillogorgiicola
CCAP84910_CONTROL_rep1_05202024	0	0	0
CCAP84910_CONTROL_rep1_05222024	0	0	0
CCAP84910_CONTROL_rep1_05242024	0	0	0
CCAP84910_CONTROL_rep2_05202024	0	0	0
CCAP84910_CONTROL_rep2_05222024	0	0	0
CCAP84910_CONTROL_rep2_05242024	0	0	0
CCAP84910_NMP1_rep1 _05202024	207	135	0
CCAP84910_NMP1_rep1_05222024	270	283	0
CCAP84910_NMP1_rep1_05242024	143	42	0
CCAP84910_NMP1_rep2_05202024	123	113	0
CCAP84910_NMP1_rep2_05222024	426	256	0
CCAP84910_NMP1_rep2_05242024	99	22	0
CCAP84910_NMP6_rep1_05202024	271	226	0
CCAP84910_NMP6_rep1_05222024	1432	718	**2**
CCAP84910_NMP6_rep1_05242024	67	59	0
CCAP84910_NMP6_rep2_05202024	480	235	0
CCAP84910_NMP6_rep2_05222024	335	152	0
CCAP84910_NMP6_rep2_05242024	65	13	0
P7C12_CONTROL_rep1_05202024	0	0	0
P7C12_CONTROL_rep1_05242024	0	0	0
P7C12_NMP1_rep1_05202024	137	149	**2**
P7C12_NMP1_rep1_05242024	0	0	0
P7C12_NMP6_rep1_05202024	517	343	0
P7C12_NMP6_rep1_05242024	66	29	0

Table S4. Number of forward and reserve sequence count (either forward or reverse direction) per each sample.

Sample ID	Strain	Treatment	Replication	Day	Sequence Count
CCAP84910_CONTROL_rep1_05202024	CCAP 849/10	control	1	0	174746
CCAP84910_CONTROL_rep1_05222024	CCAP 849/10	control	1	2	153121
CCAP84910_CONTROL_rep1_05242024	CCAP 849/10	control	1	4	52568
CCAP84910_CONTROL_rep2_05202024	CCAP 849/10	control	2	0	158844
CCAP84910_CONTROL_rep2_05222024	CCAP 849/10	control	2	2	220648
CCAP84910_CONTROL_rep2_05242024	CCAP 849/10	control	2	4	145189
CCAP84910_NMP1_rep1 _05202024	CCAP 849/10	NMP1	1	0	99007
CCAP84910_NMP1_rep1_05222024	CCAP 849/10	NMP1	1	2	128376
CCAP84910_NMP1_rep1_05242024	CCAP 849/10	NMP1	1	4	137647
CCAP84910_NMP1_rep2_05202024	CCAP 849/10	NMP1	2	0	74679
CCAP84910_NMP1_rep2_05222024	CCAP 849/10	NMP1	2	2	119673
CCAP84910_NMP1_rep2_05242024	CCAP 849/10	NMP1	2	4	92075
CCAP84910_NMP6_rep1_05202024	CCAP 849/10	NMP6	1	0	75157
CCAP84910_NMP6_rep1_05222024	CCAP 849/10	NMP6	1	2	191248
CCAP84910_NMP6_rep1_05242024	CCAP 849/10	NMP6	1	4	180795
CCAP84910_NMP6_rep2_05202024	CCAP 849/10	NMP6	2	0	164038
CCAP84910_NMP6_rep2_05222024	CCAP 849/10	NMP6	2	2	129807
CCAP84910_NMP6_rep2_05242024	CCAP 849/10	NMP6	2	4	38521
P7C12_CONTROL_rep1_05202024	P7C12	control	1	0	164409
P7C12_CONTROL_rep1_05242024	P7C12	control	1	4	72706
P7C12_NMP1_rep1_05202024	P7C12	NMP1	1	0	129084
P7C12_NMP1_rep1_05242024	P7C12	NMP1	1	4	108184
P7C12_NMP6_rep1_05202024	P7C12	NMP6	1	0	149808
P7C12_NMP6_rep1_05242024	P7C12	NMP6	1	4	89620

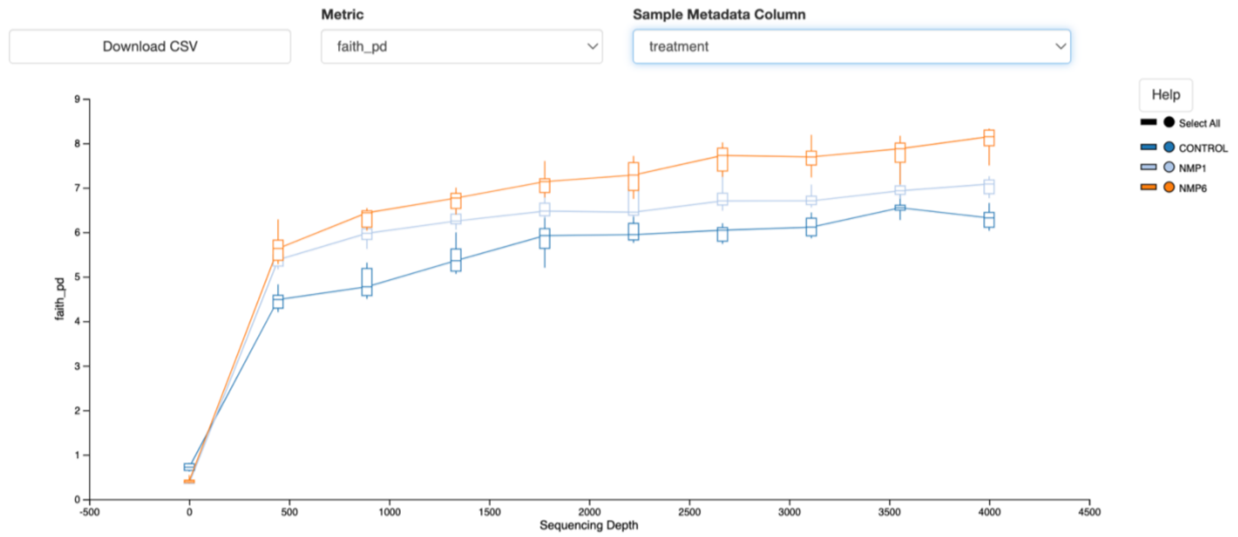

Figure S4. Alpha rarefaction plot by faith phylogenetic diversity (faith_pd) at multiple different sequencing depth. All the samples reach the stabilize point at around 4000 sequence count. All samples in this study were sequenced from ~ 40,000 to ~200,000 reads.
